# Working out dads (WOD): a study protocol for a randomised controlled trial of a group-based peer support intervention for men experiencing mental health difficulties in early fatherhood

**DOI:** 10.1186/s12888-022-03698-5

**Published:** 2022-02-12

**Authors:** Rebecca Giallo, Monique Seymour, Alison Fogarty, Casey Hosking, Le Ann Williams, Amanda Cooklin, Anneke Grobler, Jemimah Ride, Liana Leach, Brian Oldenburg, Catherine Wood, Rohan Borschmann, Jacquie O’Brien, Kirsty Evans, Karli Treyvaud, Craig Garfield, Stephanie Brown, Jan Nicholson

**Affiliations:** 1 Murdoch Children’s Research Institute, 50 Flemington Road, Parkville, Victoria 3052 Australia; 2 The University of Melbourne, Parkville, Australia; 3 LaTrobe University, Bundoora, Australia; 4 Tweddle Child & Family Health Service, Footscray, Australia; 5 The Australian National University, Canberra, Australia; 6 Baker Heart & Diabetes Institute, Melbourne, Australia; 7 Swinburne University of Technology, Hawthorn, Australia; 8 Northwestern University, Evanston, IL USA

**Keywords:** Fathers, Mental health, Peer support intervention, Early parenthood, Randomised controlled trial, Effectiveness, Cost-effectiveness

## Abstract

**Background:**

Approximately one in ten men experience mental health difficulties during the early years of fatherhood, and these can have negative impacts on children and families. However, few evidence-based interventions targeting fathers’ mental health are available. The aim of the trial is to evaluate the effectiveness and cost-effectiveness of Working Out Dads (WOD) – a facilitated peer support group intervention for fathers of young children, in reducing psychological distress and other mental health symptoms.

**Methods:**

This trial will employ a parallel-arm randomised controlled trial (RCT) to evaluate the effectiveness and cost effectiveness of WOD peer support group intervention compared to usual care (a 30-min mental health and service focused phone consultation with a health professional). A total of 280 fathers of young children (aged 0-4 years) who are experiencing mental health difficulties and/or are at risk of poor mental health will be recruited.

Randomisation and analyses will be at the level of the individual participant. The primary outcome is psychological distress symptoms, measured by the Kessler Psychological Distress Scale (K10) from baseline to 24 weeks post randomisation. A range of secondary outcomes will be assessed including suicidal ideation; mental health disorders, specific symptoms of depression, anxiety, and stress; social support, quality of life, health service use, and health care costs. Data will be collected at baseline, 10- and 24 weeks post-randomisation.

**Discussion:**

This trial will examine the effectiveness of a novel group-based peer support intervention in reducing the psychological distress and other mental health symptoms of fathers compared to usual care. The economic and process evaluation will guide policy decision making along with informing the future implementation of WOD on a larger scale if effectiveness is demonstrated.

**Trial registration:**

The current trial has been registered with ClinicalTrials.gov (Registration ID - NCT04813042). Date of Registration: March 22nd, 2021.

## Background

One in ten fathers experience mental health difficulties in the critical early years of their children’s lives [1–3]. Without effective early intervention and support, these difficulties may worsen over time for some fathers [1], have adverse consequences for families and children [4, 5], and increase risks for suicide [6]. Despite this, few evidence-based interventions to reduce mental health difficulties and suicidality in early fatherhood exist [7]. To address this gap, Working Out Dads (WOD), a facilitated peer support group intervention for fathers of young children (0-4 years) experiencing, or at risk of, mental health difficulties was developed [8]. This paper describes the protocol for a randomised controlled trial (RCT) to determine the effectiveness and cost-effectiveness of WOD in reducing mental health difficulties compared with usual care.

### Mental health difficulties and their effects in early fatherhood

It is estimated that approximately one in ten fathers experience mental health difficulties in early fatherhood. A meta-analysis of 43 studies revealed that 10% of fathers reported depressive symptoms in the pre- and postnatal periods, and that estimates were highest at 26% during the 3- to 6- month postnatal period [9]. In another review, the prevalence for an anxiety disorder in the prenatal period ranged from 4- 16% and 2-18% in the postnatal period [10]. For some fathers, mental health difficulties persist and worsen over time. Data from the Longitudinal Study of Australian Children (LSAC) revealed that approximately 30% of fathers who reported clinically significant psychological distress in the first postnatal year continued to report distress at a similar or worse level when their children were 2-3 years and 4-5 years [2]. In another longitudinal study of men from adolescence into young adulthood in the US-based Longitudinal Study of Adolescent Health, men who became fathers had 68% increase in their depressive symptom scores on average in the 5 years after becoming a father compared to age-matched non-fathers [11].

Poor father mental health may have negative consequences for children and families. Studies have reported that fathers with mental health difficulties have lower parenting self-efficacy [12] and engage in (a) less play, reading and other enrichment activities with children [13, 14], and (b) more harsh or hostile interactions with their children [5, 12]. Several studies have also shown that fathers’ mental health difficulties are associated with poor partner mental health [1], couple relationship difficulties and high levels of parental conflict [15, 16], and emotional-behavioural difficulties in children and adolescents [17–19].

Poor mental health among fathers may also heighten their risk for suicide. Although the prevalence of suicide among fathers in the early years of parenting is unknown, suicide is a leading cause of death among men aged 20-44 years in Australia [20]. This coincides with a life stage when many are raising young children. Early fatherhood is an opportune time for interventions to reduce mental health difficulties and prevent suicide [21, 22], and to prevent the potential impacts on children if they lose a father to death by suicide [23]. Although fathers have shared (in qualitative studies) that they are open to support at this time, and want to be asked about their mental health by professionals in maternity and early childhood settings, they are rarely asked about their needs and often feel they are ‘in the background’ [21, 22]. This may be due to (a) fathers not being viewed as the primary client of maternity and early childhood services, and (b) health professionals lack of skills and confidence to engage fathers about mental health concerns and refer them for interventions and support [24].

### Interventions for fathers with mental health difficulties in the early parenting period

Recent systematic reviews highlight that few evidenced-based interventions targeting men’s mental health in early fatherhood are available [7, 25, 26]. The most recent systematic review identified 14 RCTs that evaluate the effectiveness of ten individual, couple or group-based psychoeducational interventions assessing changes in fathers’ mental health in the perinatal period [7]. Positive intervention effects were found for six of the 14 studies, and the interventions with the strongest effects were those that addressed factors related to men’s lifestyle and wellbeing. This is an important consideration for the development of future interventions, given that risk factors for fathers’ mental health difficulties include having a past history of mental health problems and suicidality; relationship problems; financial and work stress; alcohol and substance use; low parenting self-efficacy, and lack of social support [1, 3, 27–30].

Other important considerations for the development of interventions are (a) men’s preferences for mental health support, and (b) how to overcome identified barriers to health service use among men. For example, male survivors of suicide report wanting practical and emotional support to manage symptoms and stress, help to focus on their family roles and responsibilities, and opportunities to connect with other men in informal settings [31, 32]. Opportunities for informal support are also important for overcoming stigma and negative attitudes to help-seeking for mental health difficulties [33], and were an important consideration for the development of Working Out Dads.

### Working out dads: a group-based peer support intervention

**Fig. 1:**
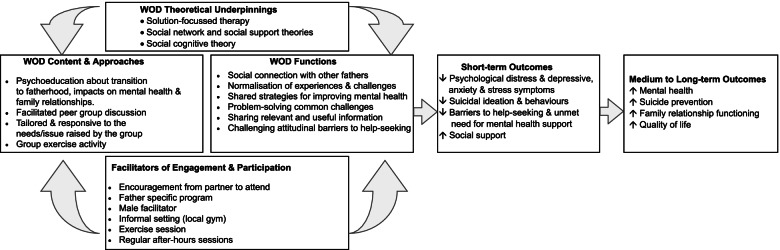
WOD Intervention logic

Working Out Dads (WOD) is a group-based peer support intervention for fathers of young children (0-4 yrs) experiencing, or at risk of, poor mental health including suicidal ideation. Figure 1 presents the intervention logic for WOD including inputs such as content and approaches, functions, theoretical underpinnings, facilitators to engagement and participation, and the hypothesised short- and longer-term outcomes. Six weekly sessions comprise a 1-h group discussion focused on common challenges and risk factors for poor mental health in early fatherhood, followed by a 30-min group exercise session provided by a qualified trainer. To overcome barriers to health service use mentioned above, evening sessions are held in an informal setting such as a local gym or a community setting (e.g., maternal child health centre, local council rooms, local hall, local park). The sessions are delivered by a male health professional based on current Australian evidence about fathers’ preferences for mental health care [21, 22]. Between sessions, fathers are supported in their use of WOD strategies via digital technologies (i.e. WhatsApp group) to share resources (e.g., sheets, self-monitoring tools, details of telephone helplines and other services) and facilitate peer support and social connections with members of the group.

WOD is underpinned by solution-focussed therapy and social cognitive theories, where fathers are encouraged to share practical ideas for managing stress, revisit previous successful strategies, validate what they are doing well, and explore solutions to challenges experienced. The potential for these processes to operate is particularly strong in group-based interventions grounded in social network and social support theories [34, 35]. Participants are provided with an opportunity to connect with other fathers, normalise their experiences, and challenge attitudinal barriers to help-seeking.

To date, WOD has been evaluated in feasibility and pilot research studies with fathers of young children (0-4 years) experiencing mental health difficulties and/or risk factors for poor mental health such as high work-related stress or couple relationship difficulties [36, 37]. In a service-led feasibility study with 57 fathers, there were significant pre- to post-decreases in depressive and stress symptoms associated with moderate effect sizes [36]. Following this, a non-controlled pilot study with 53 fathers included a 3-month follow-up [37]. Qualitative interviews were also conducted to explore fathers’ experiences of WOD, perceived outcomes, and barriers and facilitators to participation. A diverse group of fathers participated (~ 50% born outside Australia and high school education only) and acceptability was demonstrated as evidenced by minimal drop out (~ 10%) and 90% of participants indicating high satisfaction. At baseline 32% of fathers reported psychological distress in the Kessler Psychological Distress Scale (6 item) clinical range, which decreased to 17% at post-intervention and 6% at follow-up. There were also significant pre-post decreases in symptoms of depression, anxiety and stress, and increases in social support and parenting self-efficacy. These changes were associated with small to moderate effect sizes, and were maintained or strengthened at follow-up. The interviews with fathers revealed a range of benefits including increased health literacy, improved social support, normalisation of their experiences of early fatherhood, greater confidence in parenting, and more positive interactions with their children and partner.

Based on these promising findings, a rigorous trial will be undertaken. A longer-term follow-up at 24 weeks (6 months) will be conducted and additional outcomes included such as suicide ideation and behaviours, and a diagnostic clinical interview. A comprehensive process evaluation will guide refinement of the WOD intervention, training and resources for wider deployment in the future, if demonstrated to be effective. Importantly, an economic evaluation will assess the cost-effectiveness of WOD and the costs associated with taking this intervention to scale.

### Aims and hypotheses

The primary aim of this trial is to test the effectiveness and cost-effectiveness of WOD compared with usual care which is typically provided in community and primary health care settings (brief risk assessment and referral). We hypothesise that, compared with usual care, the WOD intervention group will have the following outcomes:

Primary outcome

1. Lower psychological distress at 24 weeks post-randomisation

Secondary outcomes

2. Lower severity of suicide ideation and behaviours at 10 and 24 weeks post-randomisation
3. Fewer specific symptoms of depression, anxiety and stress at 10 and 24 weeks post-randomisation
4. Lower proportion affected by a depressive or anxiety disorder at 24 weeks post-randomisation
5. Higher perceived social support at 10 and 24 weeks post-randomisation
6. More positive attitudes to help-seeking at 24 weeks post-randomisation
7. Lower self-reported unmet need for health services at 24 weeks post-randomisation
8. Higher parenting self-efficacy at 24 weeks post-randomisation
9. Improved parent-child relationship at 24 weeks post-randomisation.

Health economic outcomes

10. Improved quality of life at 24 weeks post-randomisation
11. Favourable cost effectiveness at 24 weeks post-randomisation

## Methods/design

### Overall study design and approach

This is a parallel-arm RCT of the WOD peer support group intervention versus usual care conducted in the state of Victoria, Australia. Randomisation and analyses will be at the level of the individual participant but the intervention will be delivered in a group context. In addition to the outcome evaluation, a health economic evaluation will be conducted to assess the cost-effectiveness of WOD. A comprehensive process evaluation will also be conducted and guided by the Reach, Effectiveness, Adoption, Implementation, Maintenance (RE-AIM) model [38], and the Exploration, Preparation, Implementation, Sustainment (EPIS) framework [39]. The process evaluation will determine the factors related to program innovation, the organisational context, and the broader system that will likely influence the successful adoption and wider implementation of WOD in the future.

The study protocol has been prepared according to the Standard Protocol Items: Recommendations for Intervention Trials (SPIRIT) statement. See Table 1 for Schedule of enrolment, interventions, and assessments as per the SPIRIT statement. The trial has been registered with ClinicalTrials.gov (Registration ID - NCT04813042), and ethics approval granted from the Royal Children’s Hospital Human Research Ethics Committee. The trial has also been approved by the Murdoch Children’s Research Institute Trials Sponsorship Committee who will review the trial every six months and conduct an audit if required. The trial will be reported in accordance with CONSORT guidelines for RCTs. The Trial Steering Committee will meet every 1-2 months to monitor the project timeline, risks, and quality assurance processes.

**Table 1:**
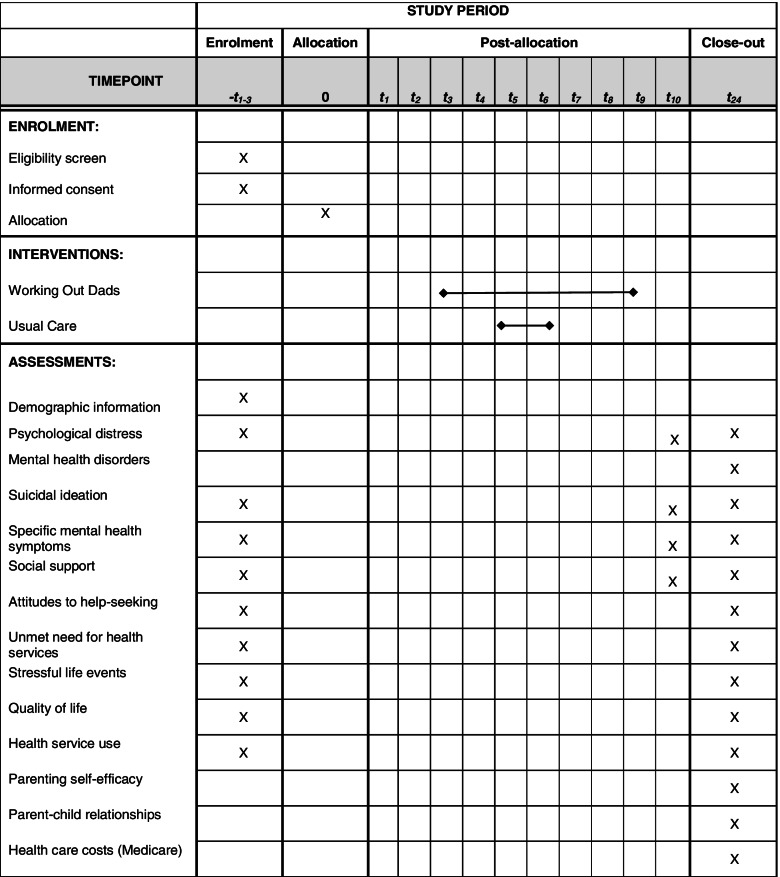
Schedule of enrolment, interventions, and assessments

### Consumer involvement and engagement

A Father Advisory Group consisting of approximately six men in early fatherhood, with or without lived experience of mental health difficulties, will be formed during the establishment of the study. Fathers attending a local child and family health service and those who participated in the WOD pilot studies will be invited to participate in the Advisory Group. They will be from diverse social and economic backgrounds to represent different cohorts of fathers in the general population. The Advisory Group will meet at least four times per year to provide input into the recruitment strategy, processes, and materials (e.g., flyers, social media posts) and other trial elements (e.g., self-report surveys, intervention resources). They will also be consulted at key stages throughout the study period to assist recruitment and retention approaches, and to assist with determining how the study findings would be best communicated to trial participants and the general population on completion. All advisory group members will be offered a $50 supermarket gift voucher following each consultation as recompense for their attendance at meetings.

### Study setting and recruitment

The participants will be fathers of young children (0-4 years) who are experiencing mental health difficulties, and/or at risk of poor mental health and suicide (see inclusion criteria below). Fathers will be recruited from four Victorian Local Government Areas (LGAs) which have a high number of births per year and young families residing there. A combination of active and passive recruitment methods will be used. Active recruitment strategies will involve research staff visiting community health settings where parents of young children frequently attend (i.e., maternal child health centres, immunisation sessions, play groups, first-time parent groups). Researchers will approach fathers and other parents/caregivers to provide them with written and/or verbal information about the study. Community health professionals (e.g., maternal child health nurses, general practitioners) will also provide fathers and/or their partners with information about the study. They may also ask for verbal consent to pass contact details on to the research team. Passive recruitment strategies will include the distribution of posters and flyers about the study in community health settings and online/social media. The recruitment materials will direct interested fathers to a webpage providing more information about the study including a narrated animation and a Project Information and Consent Form. Fathers can provide written consent electronically and begin the screening survey, or they can contact the research team if they would like to discuss the study before making a decision about participation. Additional consent will be obtained to link to administrative data on use of publicly funded healthcare services and prescriptions, Medicare Benefits Schedule (MBS) and Pharmaceutical Benefits Scheme (PBS), for health care utilisation and cost effectiveness analyses.

### Screening & inclusion/exclusion criteria

Once consent is obtained, fathers will be directed to complete the online screening survey. To be included in the trial, fathers need to meet the following criteria:

- Be a father (biological, step-, or other male caregiver) of a child age 0-4 years, and in regular weekly contact with the child
- Have mental health difficulties above the symptomatic cut-point on the Kessler Psychological Distress Scale-10 item (K10; i.e., a score of > 20, see Measures below for detail); *and/or* have at least two risk factors for trajectories of increasing mental health difficulties among men across early fatherhood [40], including: a) history or mental health difficulties; b) relationship difficulties; c) high work-related stress; d) unemployment; or e) have a child with sleep difficulties, a disability, chronic illness, or other special health care need
- Be aged 18 years or older
- Have sufficient English fluency to complete surveys and participate in the intervention.

Fathers will be ineligible to participate in the trial if they: a) have a severe mental health disorder (i.e., self-reported psychosis, substance use dependency) that may require more intensive mental health treatment; b) have an overt indicator of family violence (e.g., self-reported intervention order or court case for family violence); or c) have child protection service involvement. Following screening, the research team will contact fathers to confirm ineligibility and provide general information about mental health support available in the community (i.e., family doctor, online resources, telephone helplines).

### Sample size

A sample size of 14 peer groups per study arm with 9 individuals per group has 80% power to detect a difference of 0.4 (Cohen’s d) between standardised K10 group mean scores when the intracluster correlation (ICC) is 0.02 using a 2-sided t-test at the 0.05 significance level. The number of participants per group was increased to 10 to allow for loss to follow-up of ~ 10%. This requires recruitment of 140 participants per treatment arm.

### Randomisation

Upon completion of the consent form, screening and baseline survey, fathers will be randomised in a 1:1 ratio to either WOD or the usual care (control) group (see below for descriptions). A statistician not directly involved in the analysis of the trial results will prepare the randomisation schedule using permuted block randomisation, stratified by LGA, using a computerized random number generator. The schedule will be held by the independent statistician and embedded within the web-based data management system. Treatment allocation will be concealed prior to randomisation, and only be revealed after it has been confirmed that the participant is eligible, enrolled and completed the baseline questionnaire. Once randomised, the web-based data management system will display the father’s assignment for the Project Coordinator, who will notify the father of his treatment assignment and intervention/usual care information by telephone. Due to the nature of the intervention, it is not possible to blind participants to their allocation. However, one secondary outcome is assessed by a researcher-blinded diagnostic interview of mental health disorders at the 6-month follow-up.

To minimise participant disappointment, selection bias and drop out from Usual Care, the study will be advertised as a project about comparing two different ways to promote fathers’ mental health in the early parenting period: WOD or a single telephone consultation which will be presented as convenient and potentially beneficial approach. Fathers will also be reimbursed for time taken to complete surveys (AUD$20 voucher per data collection).

### Intervention content, delivery and training

#### Content and delivery

Each session will be delivered by a facilitator to 8-10 fathers following the WOD manual. The weekly sessions will be comprised of a 1-h facilitated discussion about common challenges and risk factors for poor mental health among fathers including (a) experiences of being a father, the important role fathers have in children’s lives, play, and building relationships with children, (b) managing work-life-family balance and time for self, (c) emotional regulation and stress management, (d) coping with changes in adult relationships and strategies to maintain them, and (e) importance of health behaviours including exercise. The male health professional will facilitate participation in discussion, responds to individual needs of group members, and ensures the discussion is responsive to needs arising from the group.

A 30-min group fitness session will follow the peer group session and be tailored to the individual needs of fathers based on the qualified personal trainer’s assessment of their health and level of fitness. Between sessions, fathers will receive resources (information, tip sheets, self-monitoring tools, details of services) and peer support from other fathers in their group via a WhatsApp group. A member of the research team will monitor the WhatsApp activity, but will not participate in text messaging other than to send the WOD messages and monitor for inappropriate exchanges and safety issues (see below for section on risk and safety). At the end of the 10 weeks, the member of the research team will remove themselves from the WhatsApp group. No content from the WhatsApp group is recorded or analysed.

Intervention fidelity will be promoted and assessed in several ways. The WOD facilitators will participate in regular mentoring sessions with staff from Tweddle and the lead investigator who is a psychologist. The strengths and challenges of delivering WOD content and facilitating groups will be discussed. A session fidelity checklist will be completed each week to monitor the extent to which the WOD intervention is delivered as planned. Finally, a random selection of 30% of audio-recorded sessions will be coded by research staff using the session fidelity checklists to identify the extent to which content, activities, and group participation has occurred.

#### Training

WOD will be delivered by study-employed facilitators with allied health qualifications and training (i.e., psychology, social work, counsellor). Tweddle Child & Family Health Service and the lead investigator will train the health professionals in a 3-day workshop incorporating adult learning principles including: (a) the provision of didactic information (e.g., research into men’s mental health difficulties, theoretical underpinnings of WOD, pilot research findings for WOD); (b) group discussion (e.g., about delivering groups, group process, responding to fathers’ distress and potential safety concerns); and (c) skills practice or behavioural rehearsal of the WOD sessions. Facilitators will be provided with specific training about responding to fathers who may be come distressed, and assessing risk and safety.

### Usual care

WOD will be compared to usual care to evaluate whether it is more effective than care currently received in the community. Fathers allocated to the usual care condition will receive the clinical care typically provided to parents experiencing mental health difficulties by community health and primary health services. A health professional will conduct a brief (~ 30-min) telephone consultation to: (a) enquire about mental health symptoms and conduct a risk assessment for suicidal ideation; (b) provide referral options to telephone support services; and (c) encourage a general practitioner visit to discuss mental health care options. A fidelity checklist will be completed by the health professional to monitor delivery of usual care.

### Follow-up

Fathers will complete online self-report survey measures at 10 and 24 weeks post-randomisation (see Measures below). At 24 weeks post-randomisation, fathers will also complete a researcher-blind mental health diagnostic interview over the telephone (see Measures below). Participant retention strategies will include standardised wording for one email invite and two text and/or telephone reminders to complete surveys.

### Measures

Fathers will be asked to complete online self-report surveys using a web-based data management system at three time points: (i) at baseline (2-4 weeks prior to randomisation); (ii) 10 weeks after randomisation; and (iii) 24 weeks after randomisation. Details of the self-report surveys used to assess the primary and secondary outcomes are presented in Table 2. Fathers will also be asked to participate in a clinical interview for diagnosis of mental health disorders at 24 weeks after randomisation. This interview will be administered via telephone by an appropriately qualified research assistant who is blinded to group allocation.

**Table 2:** Study Measures

Outcome	Measure
Primary Outcome	
Mental health difficulties	Kessler Psychological Distress Scale – 10 (K10 [41]): 10 items assessing symptoms of depression and anxiety in last 4 weeks. Widely used in clinical trials for common mental health disorders, with well-established symptomatic and clinical cut-points, high specificity (0.96), and robust total classification accuracy (0.92) to identify mood and anxiety disorders [41].
Secondary Outcomes	
Mental health disorders	The Mini International Neuropsychiatric Interview (MINI): Brief 15-min structured diagnostic interview for common DSM-5 psychiatric disorders, validated for telephone administration and use in clinical trials [42]. Depressive and anxiety disorders will be assessed.
Suicidal ideation	The Suicidal Ideation Attributes Scale (SIDAS [43]): 5-item screener for the presence and severity of suicidal thoughts in the last month based on frequency, controllability, closeness to attempt, level of distress, and impact on daily functioning. Any ideation is indicative of risk for suicidal behaviour; scores > 21 indicate high risk.
Specific mental health symptoms	Depression Anxiety Stress Scale −21 (DASS-21 [44]): 21 items assessing symptoms of depression, anxiety, and stress in the past week, with higher scores indicating greater symptom severity.
Social support	The Medical Outcomes Study-Social Support Survey (MSSS [45]): 14 items assessing perceived availability of tangible, emotional, affective and positive support.
Attitudes to help-seeking	The Barriers to Help Seeking Scale (BHSS [46]): 31 items assessing attitudinal barriers to help seeking for mental health/wellbeing problems.
Unmet need for health services	Adapted Perceived need for Care Questionnaire: Modified version of the Perceived Need for Care Questionnaire [47] asking if different forms of mental health care was need, but could not be accessed.
Parenting self-efficacy	Karitane Parenting Confidence Scale (KPCS [48]): 15 items assessing parents’ perceived parenting self-efficacy or sense of competence in their parenting abilities.
Parent-child relationships	Child-Parent Relationship Scale-Short Form (CPRS): 15 items assessing parents’ perceptions of their relationship with their child [49].
Stressful life events	Adapted Pregnancy Risk Assessment Monitoring System (PRAMS [50]): 25 items asking about stressful life events experienced in the last year.
Health economic outcomes	
Quality of life	The Assessment of Quality of Life 8 dimension (AQoL-8D [51]) is a validated tool assessing quality of life impacts in economic evaluation. The AQoL-8D is particularly suited to measuring mental health aspects of quality of life.
Health service use and health care costs	Study designed self-reported type, number and cost of healthcare services accessed, and examination of linked MBS/PBS data covering 24 weeks from baseline to follow-up.

### Process evaluation

The objectives of the process evaluation are to generate evidence about how to: (a) guide refinement of the WOD intervention, training and resources, (b) inform decisions that health services need to make to adopt WOD, (c) support high quality implementation of WOD, and (d) enable sustained use and wider deployment of WOD in the future. This evaluation will be guided by the RE-AIM model [38] and the EPIS framework [39]. This approach will identify the intervention elements and eco-system (e.g., health service setting factors; policy context, policymakers) likely to influence the reach, adoption, implementation, scalability and population impact of WOD. Sources of data for the process evaluation include recruitment campaign monitoring, screening and eligibility information, participant tracking information, reflections from the study team, health services and father advisory group, and qualitative interviews with fathers who participate in WOD and usual care.

### COVID-19 pandemic safety measures and contingency planning

State government and institutional COVID-19 safety procedures and public health regulations will be adhered to at all times. This includes: (a) having current COVID-19 safety plans in place for all research and community locations (office, WOD group, active face-to-face recruitment sites), and (b) participant screening for COVID-19 symptoms, temperature testing upon arrival, maintaining physical distancing requirements, cleaning and hygiene procedures, minimising equipment sharing, and use of personal protective equipment where required. All research staff and WOD facilitators will undertake training in COVID-19 safety procedures. Any suspected or confirmed cases of COVID-19 will be reported via the sponsoring institution (Murdoch Children’s Research Institute; MCRI) incident reporting portal.

Whilst WOD has been designed for face-to-face in-person delivery, the program can be delivered via a telehealth platform in the event of a government public health directive to cease face-to-face gatherings. All fathers will be posted or emailed the required resources prior to the session/s (e.g., session handouts and materials). WOD will be delivered as intended, and the fidelity checklist will be completed by the WOD facilitator. The facilitator will be required to reflect upon and provide brief notes on the process issues of delivering online (e.g., challenges to delivering content or facilitating activities including the personal training component, quality of the interactions between fathers, level of participation by fathers). If face-to-face delivery is permitted, any remaining WOD sessions will resume face-to-face at the booked venue in the local community.

With respect to the COVID-19 pandemic and other extenuating circumstances leading to unplanned methodological, ethical and analytical challenges and changes, we will submit any protocol modifications for approval and update the trial registry. We will use the the CONSERVE (CONSORT and SPIRIT Extension for RCTs Revised in Extenuating Circumstances) statement to ensure transparency and completeness of reporting of any modifications [52].

### Risk and safety management

Risks can include: (a) risks to the safety and rights of the study participants; (b) risks to the successful conduct of the study; and (c) risk to the wellbeing of WOD facilitators and research staff undertaking recruitment and data collection. A trial-specific risk and safety assessment and response plan has been conducted by the Trial Steering Committee. In brief, the Principal Investigator will be notified of any safety or adverse concerns to assess severity, seriousness, and likelihood of the event to the trial conditions. These will be discussed at the Trial Steering Committee or the HREC as soon as possible to determine action (e.g., withdrawal, referral).

A comprehensive plan to minimise and respond to any psychological distress or safety issues for the study participants has been approved by HREC. This includes but is not limited to: (a) clear information in the Participant Information and Consent Form about why the study is being conducted, what it will involve, the potential risks and burdens of participation, and the opportunity to opt out of the study at any time; (b) follow-up and psychological risk assessment for any participants who score at the clinical cut points on the mental health and suicide assessment measures; (c) responding to distress in WOD or Usual Care conditions; and (d) monitoring and responding to distress or inappropriate exchanges on the WhatsApp group.

To maintain wellbeing of research staff and facilitators, training in responding to participant distress (as described above), comprehensive fieldwork guidelines, and regular supervision will be provided by the lead investigator who is a trained psychologist. Regular group supervision will also provide opportunities to process concerns in responding to participant distress or safety concerns, as well as monitoring staff wellbeing.

### Data management

All study data will be collected and managed using a secure web platform, online electronic data capture tools, hosted at MCRI. All personal and identifying information will be kept separate from trial data and linked by an ID. All data will be password protected and on secure platform at MCRI. Data will only be accessible by the research team.

### Data analysis

Primary analyses will follow the ‘intention to treat’ (ITT) principle at the level of the individual participant. In the ITT analysis participants are compared according to the group to which they were randomly allocated, regardless of participants’ compliance or withdrawal from the trial. Analysis of the primary outcome and all secondary outcomes will be done using generalised linear mixed effects models, with WOD group assigned as a random effect. The geographic location stratification variable, LGA, will be included in the model. For the primary analysis, the model based mean K10 score in each trial arm will be compared at 10 weeks and at the 6-month follow-up (primary outcome). Model-based effect estimates will be reported along with 95% confidence intervals. Binary endpoints will be analysed using generalised estimating equations, with WOD group as random effect and stratified by LGA. No interim analyses will be conducted.

### Health economics analysis

Costs of administering WOD (staff time to run groups based on number and duration of groups, training, materials, supervision, gym, travel) will be estimated drawing on the study protocol, administrative records and budgets. Analysis of costs will also include costs associated with the treatment of depression and anxiety from linked administrative data providing information on mental health care visits and prescriptions and from self-report of healthcare use. Quality of life between groups will be compared using AQoL-8D scores at baseline and 6-month follow-up. Self-reported impacts of poor mental health on employment and lost time from work will also be analysed to assess productivity impacts. The incremental cost-effectiveness ratio at the 6-month follow-up period will be calculated in terms of cost per quality-adjusted life year (QALY) and cost per primary outcome (K10) change. If there are significant differences in outcomes between control and treatment groups at follow-up, a decision analytic model will be used to extend the analysis to long-term impacts by modelling cost-effectiveness drawing on existing depression long-term simulation models. We will estimate the public healthcare budget impact of WOD under different scales of roll-out, including roll-out in Early Parenting Centres in Victoria and potentially Australia. Costs and outcomes will be discounted at 5%, and sensitivity analyses will be performed to determine the impact of changes to key parameters. The model will be extended to take into account the distribution of costs and benefits across the population to determine the impact of the intervention on equity.

## Discussion

This study will determine whether a facilitated peer support group intervention for fathers of young children experiencing mental health difficulties leads to a reduction in psychological distress and other mental health symptoms (depression, anxiety, stress and suicidal ideation) compared with usual care. It will also assess whether the group intervention leads to improvements in help-seeking for mental health concerns, social support and family relationship outcomes. The process and economic data will inform policy decision making, workforce development, scalability and the future implementation of WOD in early parenting and childhood settings. Given that the trial will be conducted during the COVID-19 pandemic, flexibility in delivery of WOD using a telehealth platform may be required. All modifications to the trial methods and intervention delivery will be reported with transparency using the CONSERVE statement for reporting trial modifications due to the COVID-19 pandemic and any other extenuating circumstances. The findings will be disseminated widely via peer reviewed journals, scientific conferences, professional associations (e.g., psychology, psychiatry, early parenting, early childhood), policy briefs for policymakers and government departments, and roundtable forums for health professionals. This is a critical step toward improving opportunities for early and effective provision of mental health care to fathers experiencing, or at risk of, mental health difficulties in the critical early years of their children’s lives.

## Data Availability

Not applicable.

## Abbreviations

CONSERVE: CONSORT and SPIRIT Extenstions for RCTs Revised in Extenuating Circumstances
CONSORT: Consolidated Standards of Reporting Trials
EPIS framework: Exploration, Preparation, Implimentation, Sustainment framework
ICC: Intracluster correlation
ITT: Intention to treat
LGA: Local government area
MCRI: Murdoch Children’s Research Institute
QALY: Quality-adjusted life year
RCT: Randomised control trial
RE-AIM model: Reach, Effectiveness, Adoption, Implimentation, Maintenance model
SPIRIT: Standard Protocol Items: Recommendations for Intervention Trials
WOD: Working Out Dads
